# Fastest predators in the plant kingdom: functional morphology and biomechanics of suction traps found in the largest genus of carnivorous plants

**DOI:** 10.1093/aobpla/plv140

**Published:** 2015-11-24

**Authors:** Simon Poppinga, Carmen Weisskopf, Anna Sophia Westermeier, Tom Masselter, Thomas Speck

**Affiliations:** 1 Plant Biomechanics Group, University of Freiburg, Botanic Garden, Schänzlestrasse 1, 79104 Freiburg im Breisgau, Germany; 2 Freiburg Materials Research Center (FMF), University of Freiburg, Stefan-Meier-Straße 21, 79104 Freiburg im Breisgau, Germany; 3 Present address: Department of Biomaterials, Max Planck Institute of Colloids and Interfaces, Wissenschaftspark Potsdam-Golm, Am Mühlenberg 1, 14476 Potsdam, Germany

**Keywords:** Biomechanics, bladderwort, carnivorous plant, functional morphology, prey, suction trap, *Utricularia*

## Abstract

Understanding the physics of plant movements, which describe the interplay between plant architecture, movement speed and actuation principles, is essential for the comprehension of important processes like plant morphogenesis. Recent investigations especially on rapid plant movements at the interface of biology, physics and engineering sciences highlight how such fast motions can be achieved without the presence of muscles, nerves and technical hinge analogies. The suction traps (bladders) of carnivorous bladderworts (*Utricularia* spp., Lentibulariaceae, Lamiales) are considered as some of the most elaborate moving structures in the plant kingdom. A complex interplay of morphological and physiological adaptations allows the traps to pump water out of their body and to store elastic energy in the deformed bladder walls. Mechanical stimulation by prey entails opening of the otherwise watertight trapdoor, followed by trap wall relaxation, sucking in of water and prey, and consecutive trapdoor closure. Suction can also occur spontaneously in non-stimulated traps. We review the current state of knowledge about the suction trap mechanism with a focus on architectonically homogeneous traps of aquatic bladderwort species from section *Utricularia* (the so-called ‘*Utricularia vulgaris* trap type’). The functional morphology and biomechanics of the traps are described in detail. We discuss open questions and propose promising aspects for future studies on these sophisticated ultra-fast trapping devices.

## Abstract (precis)

How can plants move without muscles, nerves and technical hinge analogies? Carnivorous bladderworts (*Utricularia* spp., Lentibulariaceae) perform one of the fastest movements known in the plant kingdom by capturing their prey (mainly small crustaceans) with suction traps. Capture lasts only half a millisecond, and animals are sucked into the trap with an acceleration of 600 *g*, which leaves no chance of escape. We review the current state of knowledge about these sophisticated trapping devices, highlight their biomechanical, functional-morphological and physiological peculiarities and discuss open questions for possible future studies.

## Introduction

Carnivorous plants attract, catch, retain and kill prey animals and absorb the nutrients resulting from digestion (Darwin 1875; Lloyd 1942). This ‘carnivorous syndrome’ has evolved several times independently in angiosperms and can be regarded as an adaptation to a life in nutrient-poor habitats (Juniper *et al*. 1989; Albert *et al*. 1992; Barthlott *et al*. 2007). Carnivorous plants are termed ‘active’ when their traps perform motion, as, for example, the slow movements of *Drosera* (sundew) leaf blades to retain prey. Apart from the classical textbook division into taxes, tropisms, nastic and autonomous motions, such plant movements can also be described according to their actuation principle. Hydraulic motions function due to a displacement of water between cells and tissues, which can be active (turgor changes in living cells) or passive (swelling/shrinking processes of dead cells, cohesion-force driven motion). The speed of hydraulic movement primarily depends on the dimension (thickness) of the respective plant organ which the water has to flow through and, hence, is ultimately limited by the speed of this process of water diffusion (Skotheim and Mahadevan 2005).

Some active carnivorous plants have evolved traps that can move faster as theoretically possible due to pure hydraulics (reviewed by Forterre 2013; Poppinga *et al*. 2013*a*). A well-known example for this phenomenon is the snap-trap of the Venus flytrap (*Dionaea muscipula*, Droseraceae), which performs a combination of stimulus-triggered, active hydraulic motion followed by a passive release of elastic energy stored in the trap lobes (snap-buckling) (Forterre *et al*. 2005). Such elastic components greatly boost the overall speed of the motion, which otherwise would be too slow for the carnivorous plant to overcome prey. The understanding of such mechanical ‘tricks’ not only leads to a deepened understanding of the ecology and evolution of a plant and its trapping mechanism (Gibson and Waller 2009; Poppinga *et al*. 2013*b*), but can also give great inspiration for implementation into bio-inspired technical materials (reviewed by Guo *et al*. 2015).

The recent proof of carnivory in *Philcoxia* with belowground sticky traps (Pereira *et al*. 2012), the discovery of ancient sticky trap fragments in Eocene Baltic amber (Sadowski *et al*. 2015) and comprehensive analyses of passive-dynamic prey capture mechanisms (Bauer *et al*. 2015) demonstrate that carnivorous plants are always good for ‘a surprise’. In this review, we summarize the current state of knowledge about the fastest active trapping mechanism known, the suction trap, which is far from being completely understood. We believe that it also holds ready ‘scientific surprises’ and hope to inspire future research on these still enigmatic and mechanically highly complex devices.

## Carnivory in the Lentibulariaceae

Within the flowering plant family Lentibulariaceae (order Lamiales), three carnivorous genera with different prey capture mechanisms exist. *Genlisea* (corkscrew plants) feature sub-terrestrial eel-traps (Darwin 1875; Lloyd 1942; Fleischmann 2012*a*), *Pinguicula* (butterworts) develop active sticky leaves (Darwin 1875; Lloyd 1942; Heslop-Harrison 1970) and *Utricularia* (bladderworts) capture and digest small prey animals with active suction traps (Darwin 1875; Treat 1875; Lloyd 1942; reviewed by Guisande *et al*. 2007). The family name can be deduced from the Latin word for ‘lentil’ (lens), referring to the lentiform traps of *Utricularia*, whereas the bladderwort's genus name can be ascribed to the term ‘utriculus’, which refers to the shape of a wineskin.

Bladderworts constitute the largest genus of carnivorous plants and comprise ∼240 species (Taylor 1989; Fleischmann 2012*b*, 2015). Molecular phylogenetic reconstructions showed that *Pinguicula* holds a basal position in the Lentibulariaceae and that *Genlisea* and *Utricularia* are more derived sister genera (Müller *et al*. 2000, 2004, 2006; Müller and Borsch 2005; Fleischmann 2012*a*). The aquatic *U. gibba* possesses one of the smallest angiosperm genomes so far known (only rivalled by some species of *Genlisea*) (Greilhuber *et al*. 2006; Fleischmann *et al*. 2014; Veleba *et al*. 2014), which is furthermore characterized by only a tiny portion of non-coding DNA (Ibarra-Laclette *et al*. 2013). Taylor (1989) classified 35 sections within *Utricularia* according to morphological traits, including trap shape, position of trap entrance and door, and position and shape of trap appendages. The molecular systematic analyses by Jobson *et al*. (2003), Müller *et al*. (2004) and Müller and Borsch (2005) generally corroborate this classification, and the three subgenera *Polypompholyx*, *Utricularia* and *Bivalvaria* have been proposed (Müller *et al*. 2006). The sections *Utricularia* and *Vesiculina* (*U*. subgen. *Utricularia*) comprise nearly all aquatic bladderworts, and the 35 species in section *Utricularia* share a common trap architecture (the ‘*Utricularia vulgaris* trap type’) (Lloyd 1935, 1942; Taylor 1989) that will be described in detail with all its structural and functional variations in this article. Biophysical investigations on *Utricularia* have been conducted for the most part on this trap type, as the respective aquatic species possess relative large traps and are comparably easy to access and cultivate.

## Distribution and Life-forms of *Utricularia*

*Utricularia* can be found almost worldwide, with hotspots of diversity in South America and Australia (Taylor 1989). Bladderworts occur rarely in arid regions as they need at least seasonal humidity to thrive. The widest distribution is shown by some aquatic or semi-aquatic species that can be found in the entire circumboreal region (Lloyd 1942; Taylor 1989; Barthlott *et al*. 2007).

Bladderworts grow in diverse habitats, all being characterized by soils or water poor in nutrients and sparse competition. According to their habitat, species can be divided into several life-forms, whereas the boundaries between these life-forms are often vague and intermediate forms exist (Brewer-Carias 1973; Van Steenis 1981; Taylor 1989; Müller and Borsch 2005; Reifenrath *et al*. 2006; Barthlott *et al*. 2007). Terrestrial species grow on wet soils, e.g. in constantly wet peat or in sand savannah communities where seasonally no surface water is visible. The soil has to be wet at least in the growth periods of the plants, but waterlogged soils are preferred by most species. If growing on banks, the plants can become temporarily submersed but remain anchored to the ground. This group contains more than half of all known *Utricularia* species (e.g. *U. prehensilis*, *U. trichophylla* and *U. uliginosa*). Facultative epiphytic (e.g. *U. alpina*) and facultative lithophytic species (e.g. *U. sandersonii*) can be found growing, for example, on tree trunks or on wet rocks, respectively. Aquatic species grow in more or less oligotrophic waters, either free-floating (e.g. *U. vulgaris*) or anchored submersed (e.g. *U. intermedia*). In the latter case, the plants are affixed to the ground with modified root-like shoots or make use of specialized anchoring devices for a life in vastly streaming water (rheophytes) (e.g. *U. rigida*). The classification of anchored submersed species as aquatic life-forms is not supported by all authors who classify them as semi-aquatic or semi-terrestrial. Phytotelmatic bladderworts grow in bromeliad cisterns that act as drain-off free water storages (e.g. *U. humboldtii*).

## General Morphology

Bladderworts are mostly small, herbaceous, annual or perennial plants. Most species do not reach overall lengths >30 cm but, as exceptions, some aquatic species such as *U. vulgaris* can reach a length of up to 2.5 m. The basic cormophyte organs, leaf and stem, cannot be clearly distinguished, and roots are completely absent (von Goebel 1889; Lloyd 1942; Troll and Dietz 1954). Some aquatic bladderworts develop dimorphic shoots (Friday 1991; Adamec 2007*a*).

**Figure 1.**
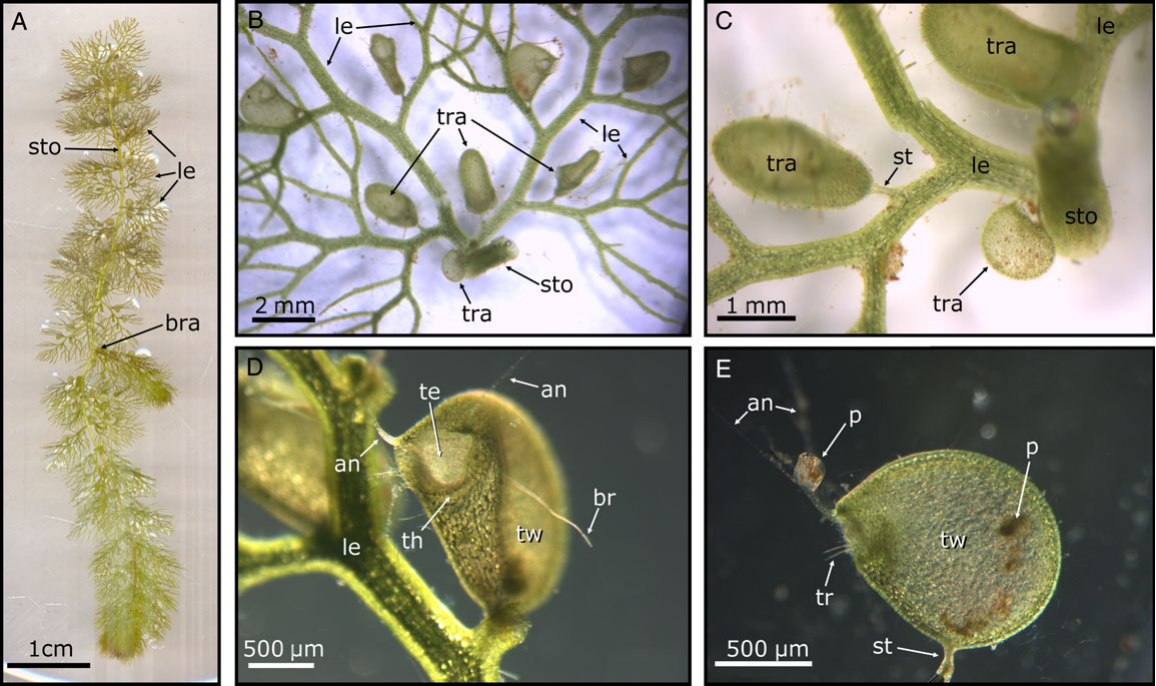
General bladderwort morphology, depicted exemplarily by *U. vulgaris*. (A) Young plant, resprouting from hibernation. The stolon (sto), leaves (le) and a branching point (bra) are clearly visible. (B) View of a detached leaf foliar shoot node featuring a trap (tra) dimorphism. Note the stolon remnant and a small, morphologically divergent trap. ‘Normal’ traps are dispersed on the pinnate leaves. (C) Detailed view of the leaf base, note the trap stalk (st). (D) Inclined frontal view of a trap. The trap entrance (te) possesses a door, a threshold (th), ‘antennae’ (an) and ‘bristles’ (br). The lateral trap wall (tw) is concave. Hence, the trap has generated underpressure inside and is ready to capture prey. (E) Lateral view of a detached trap, the entrance faces towards the left-hand side. A small prey animal (p), presumably *Chydorus* spec., grazes algae on the ‘antennae’. Already caught prey is visible inside the trap. The trigger hairs (tr) protrude from the trapdoor.

In most bladderworts, the stem is elongated and termed a stolon (Fig. 1A). This feature is absent in some phylogenetically early-branching species (e.g. *U. multifida*). Stolons are often glabrous or carry a multitude of glands. In terrestrial, facultative epiphytic and facultative lithophytic species, the stolons are very thin, only a few centimetres long and form a dense network in the soil. In aquatic species, the stolons are much thicker and longer, split up and form a characteristic branching architecture (Sattler and Rutishauser 1990; Rutishauser 1993).

Leaves are developed in rosettes, whorls or dispersed all over the stolons. In aquatic species, the classification as a ‘leaf’ is often difficult, as these species often possess leaf-like shoots (Taylor 1989). Nonetheless, the strongly branched structures emerging from the stolons of these species are termed as pinnate, filiform leaves (Juniper *et al*. 1989; Taylor 1989; Barthlott *et al*. 2007) (Fig. 1A–D).

Bladderworts do not possess true roots, but some species make use of root-like structures (rhizoids) for anchorage. Rheophytic bladderworts cling to rocky surfaces with specialized rhizoids that additionally possess adhesive trichomes (Van Steenis 1981; Taylor 1989).

Turions are produced when continuous growth is inhibited, e.g. by seasonally cold temperature or drought (Glück 1906; Wager 1928; Taylor 1989; Adamec and Kučerová 2013).

**Figure 2.**
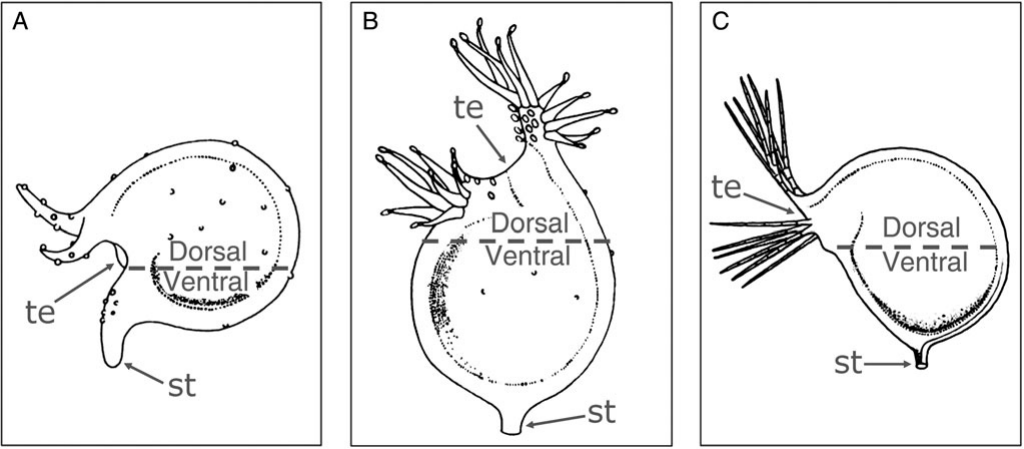
Lateral view of different trap types, indicating the position of the trap entrance (te) and of the stalk (st). (A) Basal position (*U. circumvoluta*). (B) Terminal position (*U. bisquamata*). (C) Lateral position (*U. raynalii*). The ventral and dorsal trap parts are indicated. Images modified from Taylor (1989). The genus *Utricularia*—a taxonomic monograph with kind permission from the Board of Trustees of the Royal Botanic Gardens, Kew.

The trap development takes place on different locations on the plant body, i.e. on stolons, rhizoids or leaves. In aquatic species, they mainly appear on leaves or leaf segments, at the branching points of leaves, on side shoots of stolons (e.g. *U. naviculata*) or at the leave bases (Fig. 1B–D). They are constituted of a laterally flattened hollow spherical body with a size between 0.2 mm and 1.2 cm (Taylor 1989). Rheophytic *U. neottioides* is almost completely devoid of traps (Adamec *et al*. 2015). Some species (e.g. *U. vulgaris*) feature a trap dimorphism in having two trap morphotypes that differ considerably in size (Fig. 1B and C). The bladders are connected by slender stalks to the plant body (Fig. 1B–E). The position of trap opening (also called the mouth) in relation to the point of stalk insertion varies among species: traps possessing a so-called basal position are characterized by a mouth situated directly adjacent to a stalk (Fig. 2A), a terminal position is present when the mouth is situated adversely to the stalk (Fig. 2B) and all intermediate positions are classified as lateral mouths (Fig. 2C) as it is the case in the here described *U. vulgaris* trap type (Fig. 1E). The lower trap half with the stalk insertion point is termed the ventral part, the upper half of the dorsal part (Taylor 1989) (Fig. 2). Despite their reduced chlorophyll content and low photosynthetic efficiency, the bladders, which serve for the uptake of growth-limiting plant macronutrients (nitrogen and phosphorous) from prey, are physiologically very active and require great metabolic cost (Adamec 2006).

## Prey

**Figure 3.**
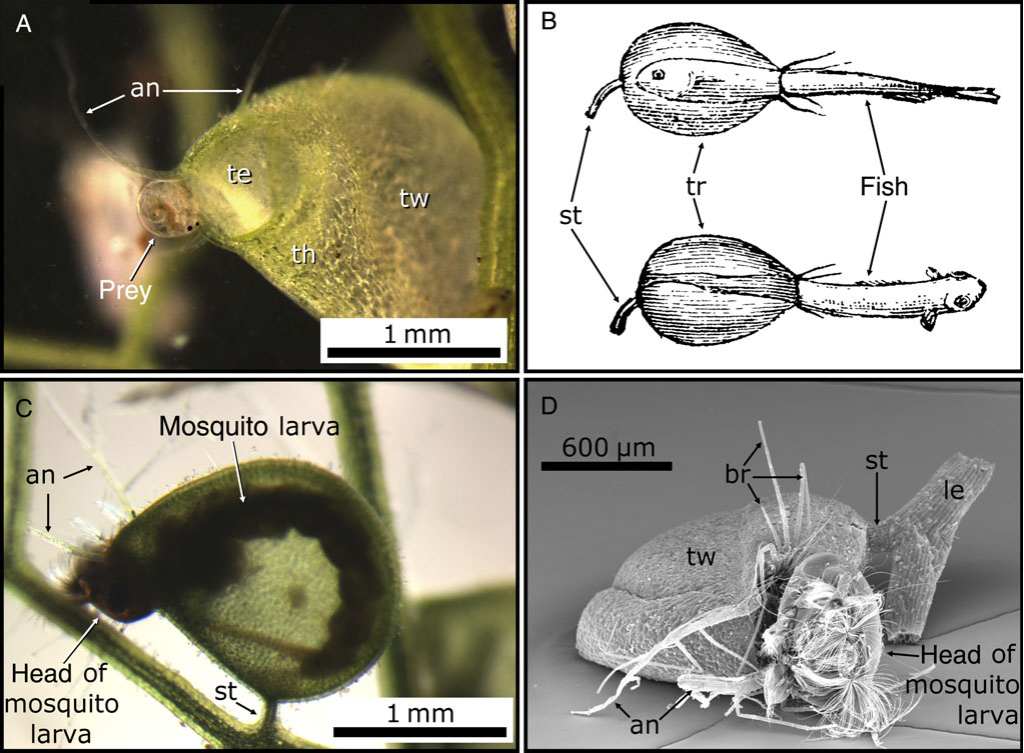
Prey of aquatic *U. vulgaris*. (A) A crustacean (probably *Chydorus* spec.) approaching the trap entrance (te); note the ‘antennae’ (an), the threshold (th) and trap wall (tw). (B) Schematic drawings of young fish as prey, head or tail first inside the traps (tr). The stalks (st) are indicated. Image modified from Gudger (1947). Reprinted with permission from AAAS. Original image adapted by permission from Macmillan Publishers Ltd: Nature (Simms 1884), copyright 1884. (C and D) Mosquito larvae as prey, tail first inside the bladder and head sticking out. (D) Scanning electron micrograph. Note also the leaf fragment (le) and trap stalk (st).

Aquatic *Utricularia* capture a wide range of members of Tardigrada, Nematoda, Gastropoda, Acaridae, Rotifera, Ciliata and Crustaceae (especially Cladocera, Copepoda and Ostracoda) (Figs 1E and 3A) (Darwin 1875; Andrikovics *et al*. 1988; Mette *et al*. 2000; Harms 2002; Sanabria-Aranda *et al*. 2006; Gordon and Pacheco 2007; Guiral and Rougier 2007; Alkhalaf *et al*. 2009; Kurbatova and Yershov 2009). Reports of large Odonata larvae (Martens and Grabow 2011), salamanders (Simms 1884) or even young fish (Moseley 1884; Gudger 1947) (Fig. 3B) as prey can most certainly be considered as exceptions. Mosquito larvae, which are also often too large for the bladders, are commonly caught tails first with their heads sticking out (Fig. 3C and D) (Brumpt 1925). Płachno *et al*. (2014) mainly found diatoms in taps of the affixed aquatic *U. volubilis*. Moreover, a multitude of other microorganisms (‘algae’, bacteria and protozoa) can be found inside the traps, which are part of complex (and not yet fully understood) food-web relationships with the plants (Hegner 1926; Schumacher 1960; Peroutka *et al*. 2008; Alkhalaf *et al*. 2009, 2011; Sirová *et al*. 2009; Płachno *et al*. 2012; Caravieri *et al*. 2014; Koller-Peroutka *et al*. 2015). For further reading on this topic, see also the section about the functional principle of the traps and the comprehensive reviews by Adamec (2011*a*, *b*) as well as the references cited therein.

In contrast, little is known about the prey spectrum in non-aquatic *Utricularia* species. Darwin (1875) found members of Rhizopoda, Crustacea and Acaridae in herbarium material; Jobson and Morris (2001) identified Insecta, Maxillopoda (especially *Elaphoidella*), Ostracoda, Branchiopoda, Chelicerata, Eutardigrada and Adenophorea (nematodes) as prey in *U. uliginosa* and Seine *et al*. (2002) discovered in laboratory experiments that Protozoa (i.e. *Blepharisma americana*) were attracted and caught by several non-aquatic *Utricularia* species.

## Functional Principle of the Traps

### Mode of functioning of traps of the *U. vulgaris* type

**Figure 4.**
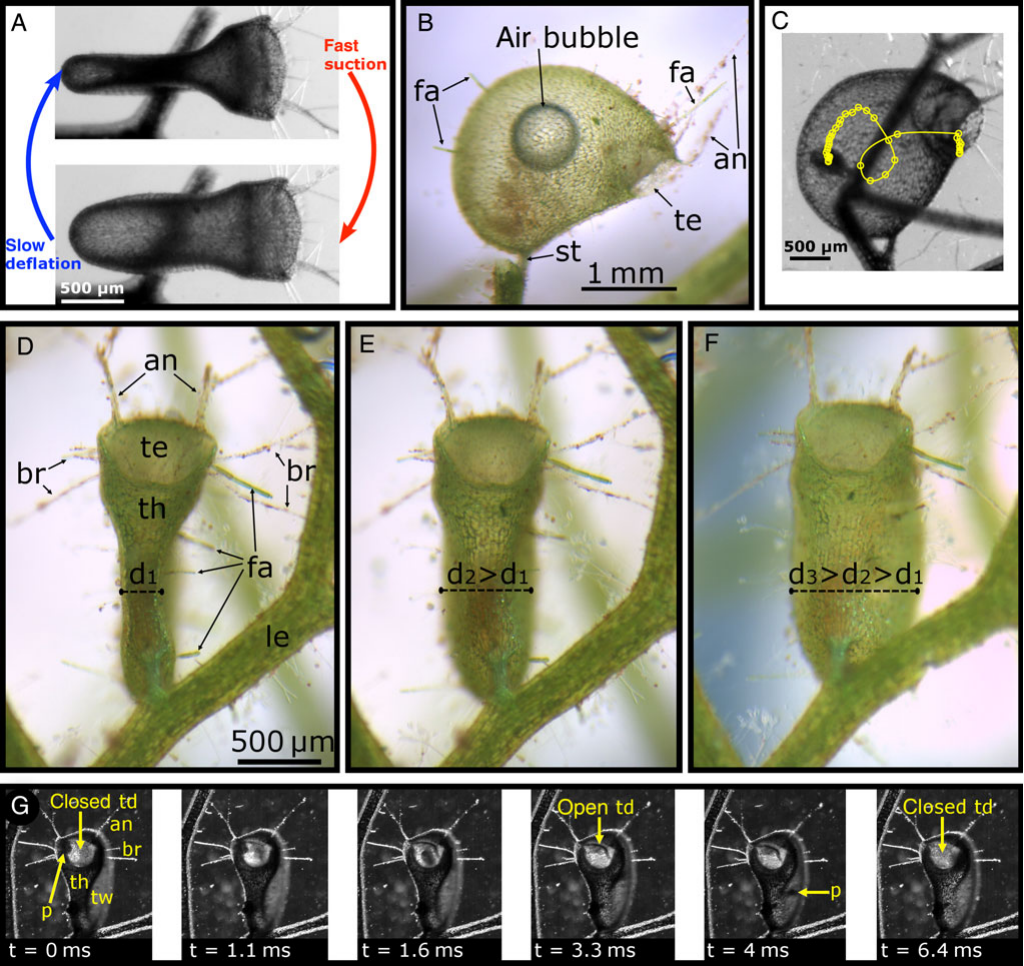
The trapping mechanism of aquatic *Utricularia*. (A) Top view of a trap which depicts the repeatable ‘active slow deflation/passive fast suction’ sequence. Note the deformation of the lateral trap walls (which store elastic energy) in the ready-to-catch condition (upper image). The species shown is *U. inflata*. (B) When traps fire in air, they aspirate air bubbles. Too large a bubble can short-circuit the deflation process. The species shown is *U. vulgaris*; note the trap entrance (te), the stalk (st), the ‘antennae’ (an) and numerous filamentous algae growing on the trap body (fa). (C) Digital tracking of prey that becomes sucked into a bladder of *U. inflata*. The animal rotates and loops inside the trap. (D) A fully deflated trap of *U. australis* in the ready-to-catch condition; note the leaf (le) and ‘bristles’ (br) on the trap. The trap diameter (d1) between the lateral trap walls is indicated. (E) The same trap after firing (by manual triggering with a fine nylon thread). The trap diameter (now d2) has increased owing to the more or less relaxed trap walls. (F) After piercing the trap with a fine needle (hole not visible), the trap diameter (now d3) has further increased, which indicates that in (E), the pressure difference between the inside and outside of the trap was not yet completely levelled. (D–F) have identical scale bars. (G) High-speed video frames (recording speed: 10 000 fps) of a prey (p) capture event in *U. vulgaris*. The first image, which shows a fully deflated trap in the ready-to-catch state seen in an inclined frontal view, correlates with (D). The prey animal, presumably *Chydorus* spec., triggers the trapdoor (td), which opens (note also the threshold (th)). After 1.1 ms, the prey begins to swirl into the trap. After 3.3 ms, the door starts moving back until at 6.4 ms it is fully reclosed. At this time, the trap state corresponds to (E) and the lateral trap walls (tw) are still concave. This indicates that there is still a pressure difference between the inside and the outside of the trap and that the door has closed during the influx of water. (A and C) Modified from Vincent *et al*. (2011*b*).

The aquatic *Utricularia* trap works in two distinguishable phases (Fig. 4A). It represents a hollow vesicle filled with water, which is, in the first phase, actively pumped out of the trap body by specialized glands (see also the section about the functional morphology of traps of the *U. vulgaris* type). Adaptive changes in the transmembrane protein complex cytochrome *c* oxidase provide respiratory power for this energy-demanding process that induces a lower internal hydrostatic pressure in respect to the outer medium (Sydenham and Findlay 1975; Jobson *et al*. 2004; Laakkonen *et al*. 2006). Sasago and Sibaoka (1985*a*) determined a pressure difference of 0.14 bar in *U. vulgaris*, and Singh *et al*. (2011) measured a difference of 0.12 bar in *U. stellaris*. Owing to the pressure difference and the resulting underpressure inside the bladder, a ready-to-catch trap shows concave curvatures (as seen from the outside) of its lateral, flexible trap walls, which by this store elastic energy (Figs 1D, 3A and 4). Large traps can easily be manipulated manually to reset to a deflated state by pressing the lateral trap walls, hereby squeezing water out of the trap through the entrance. In traps taken out of the water that fire in air, which is accompanied by an audible clicking noise (Clark 1880), the deflation process may become short-circuited owing to air bubbles (Fig. 4B) (Adamec 2012).

**Figure 5.**
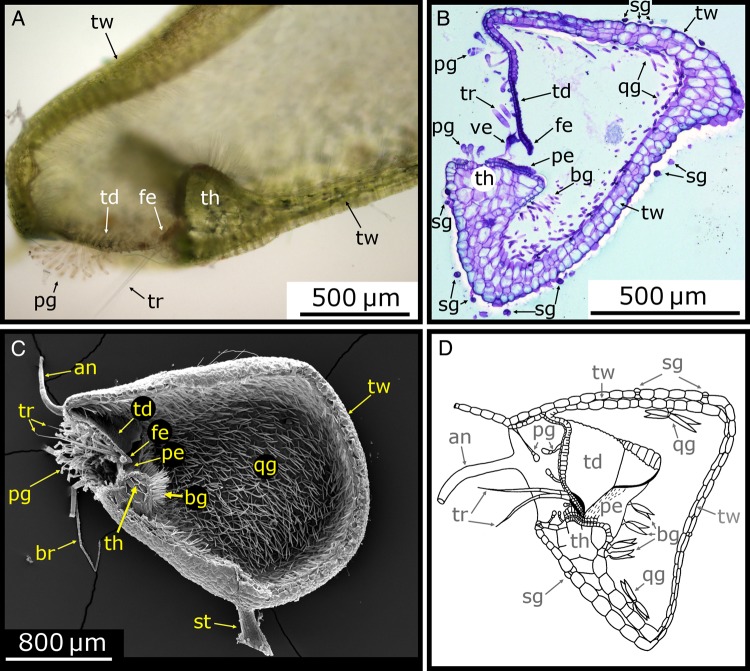
Morphology of the trap body. (A) Light microscope (LM) image of a longitudinal section of a *U. vulgaris* trap cut open with a razor blade. The door (td) with its free edge (fe), the threshold (th), trigger hairs (tr), pyriform glands (pg) and the trap wall (tw) are visible. (B) LM image of a 10-µm-thick semi-thin longitudinal section of a *U. vulgaris* trap, stained with toluidine blue. The trap wall, trapdoor, threshold, spherically headed glands (sg), bifid gland (bg), quadrifid gland (qg) and the velum (ve) can be seen. (C) Scanning electron microscope image of a longitudinal section of a *U. vulgaris* trap (cut with a razor blade before critical point drying). Among the many structures situated at the trap entrance, especially the pavement epithelium (pe) and the bifid and quadrifid glands covering the inside of the trap are noteworthy. The stalk (st), ‘antennae’ (an) and ‘bristles’ (br) are also visible. (D) Schematic drawing of a longitudinal section of a *U. gibba* trap. Image modified from Lloyd (1932) (© Canadian Science Publishing or its licensors). Note that in all images, the trapdoor is arranged at an ∼90° angle to the threshold surface, which is characteristic for the *U. vulgaris* trap type.

**Figure 6.**
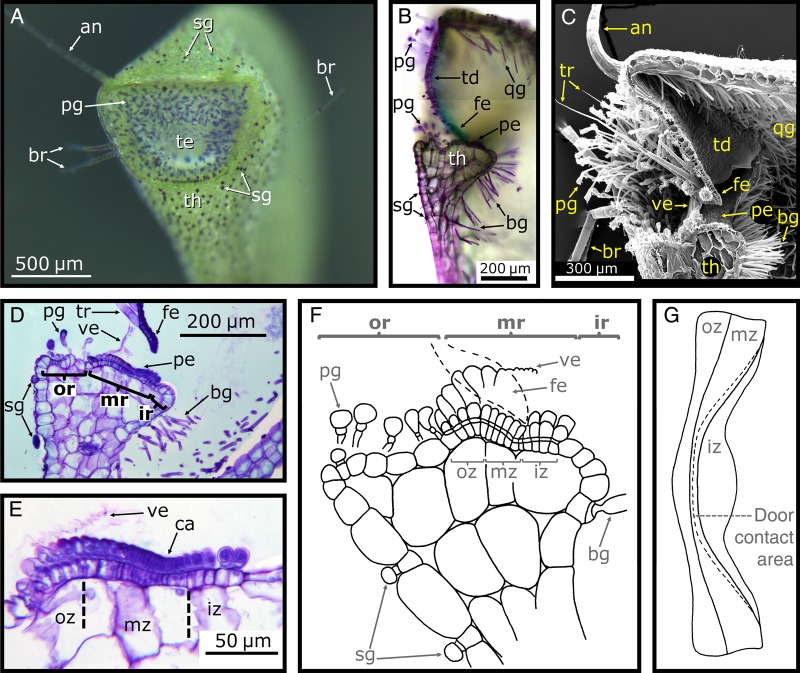
Trap entrance and compartments of the threshold. (A) Inclined frontal view of a *U. vulgaris* trap entrance (te). After dipping the trap into toluidine blue for a few minutes, the spherically headed glands (sg) covering the outer trap surface and threshold (th) and pyriform glands (pg) at the trap entrance are very well visible. The ‘antennae’ (an) and ‘bristles’ (br) can also be seen. (B) LM image of a longitudinal section of a *U. vulgaris* trap entrance (cut with a razor blade), stained with toluidine blue. Note the trapdoor (td) with its free edge (fe), the pavement epithelium (pe), the bifid gland (bg) and the quadrifid gland (qg). (C) Scanning electron microscope image of a longitudinal section of a *U. vulgaris* trap entrance (cut with a razor blade before critical point drying); note the velum (ve). (D) LM image of a 10-µm-thick semi-thin longitudinal section of the threshold. The free door edge is also visible; note the outer (or), middle (mr) and inner (ir) region. (E) LM image of a 10-µm-thick semi-thin longitudinal section of the pavement epithelium showing the outer (oz), middle (mz) and inner (iz) zone. The door contact area, the cavity (ca), is well visible. (F) Schematic drawing of a longitudinal section of the threshold of *U. gibba*. The free door edge is depicted by dashed lines and is in contact with the velum, as well as with the pavement epithelium (in the cavity). (G) Schematic top view of the pavement epithelium, showing the different zones and the contact area with the door along the cavity. (F and G) Modified from Lloyd (1936*a*) (© Verlag Heinrich Dresden, reproduced from the Digital Library of the Royal Botanical Garden of Madrid (CSIC)).

**Figure 7.**
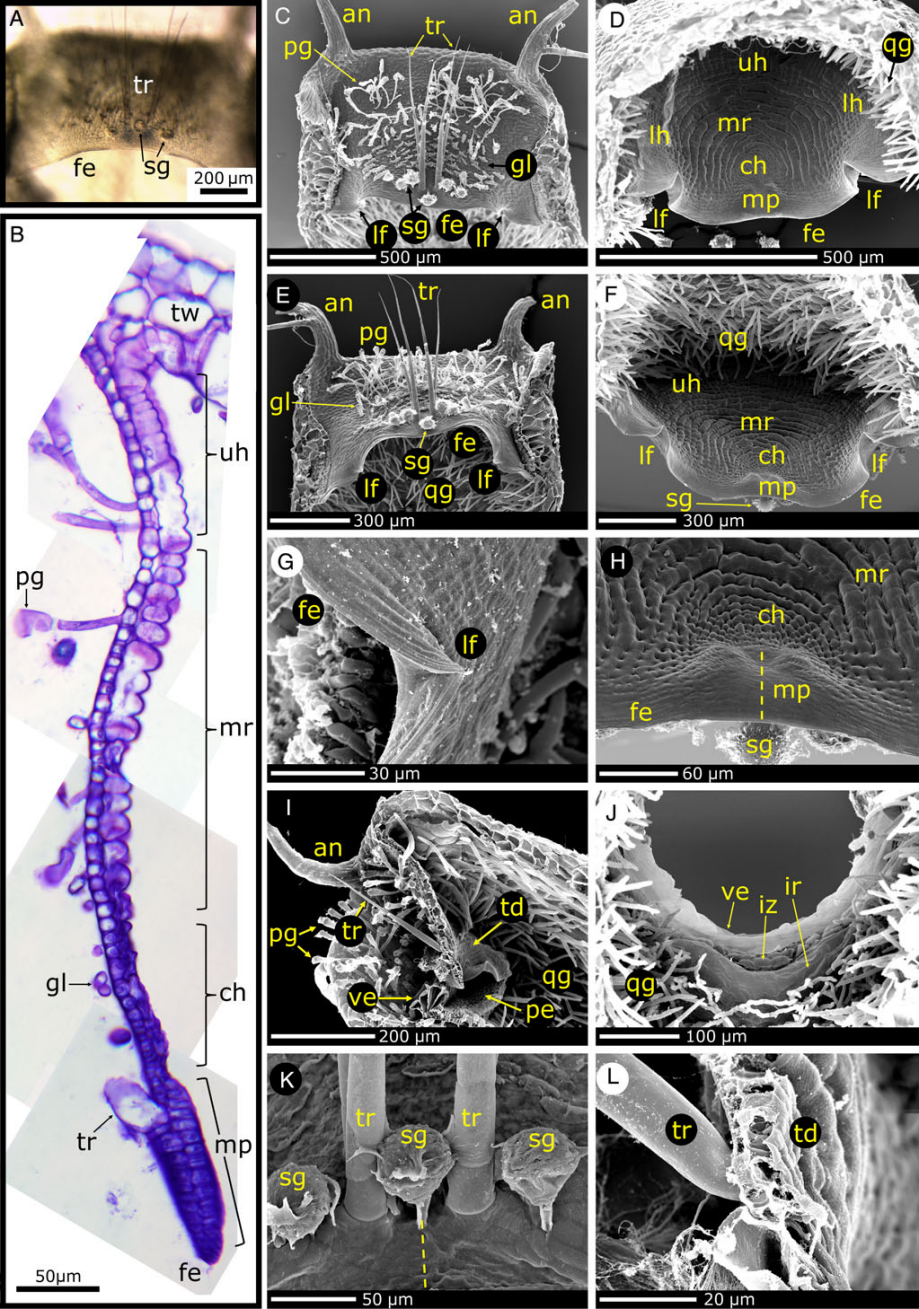
Functional morphology of the trapdoor (species shown: *U. vulgaris*). (A) A dissected trapdoor. Note the free door edge (fe), trigger hairs (tr) and spherically headed glands (sg). (B) Composite LM image of a 10-µm-thick semi-thin longitudinal section of a trapdoor connected to the trap wall (tw). The trapdoor compartments (upper hinge (uh), middle region (mr), central hinge (ch), middle piece (mp)) and structures at the outer surface of the trapdoor (on the left-hand side) (trigger hairs (tr), glands (gl), pyriform glands (pg)) are indicated. (C–L) Scanning electron microscope images of trapdoor regions. (C) Trap entrance with ‘antennae’ (an) and outer trapdoor surface with trigger hairs (tr) and various glands; note also the lateral folds (lf) on the free edge of the trapdoor. (D) Inner trapdoor surface with the conspicuous cellular arrangements depicting the different door regions. Quadrifid glands (qg) are also visible. (E) Oblique view of the outer trapdoor surface. (F) Oblique view of the inner trapdoor surface. (G) Detailed view of a lateral trapdoor fold. (H) Detailed view of the central hinge and middle piece on the inner trapdoor surface. Note the bulges on the middle piece and central hinge. Between these bulges, which are also visible on the outer trapdoor surface, the trapdoor is very thin (indicated by a dashed line) and can easily deform, which causes collapsing of the trigger hairs during trapdoor movement. The spherically headed glands at the area of the trigger hairs insertion on the outer trapdoor surface are also visible. (I) Image of a longitudinal section of a trap entrance. The trapdoor (td) and velum (ve) are well visible. (J) Detailed view of the velum. The inner zone (iz) and inner region (ir) on the threshold are also visible. (K) Area of trigger hair insertion with spherically headed glands. The dashed line indicates according to (H) the area between the bulges where the trapdoor is very thin. (L) Longitudinal section of the trapdoor area where a trigger hair inserts into the trapdoor. (C and D) Modified from Vincent *et al*. (2011*b*).

**Figure 8.**
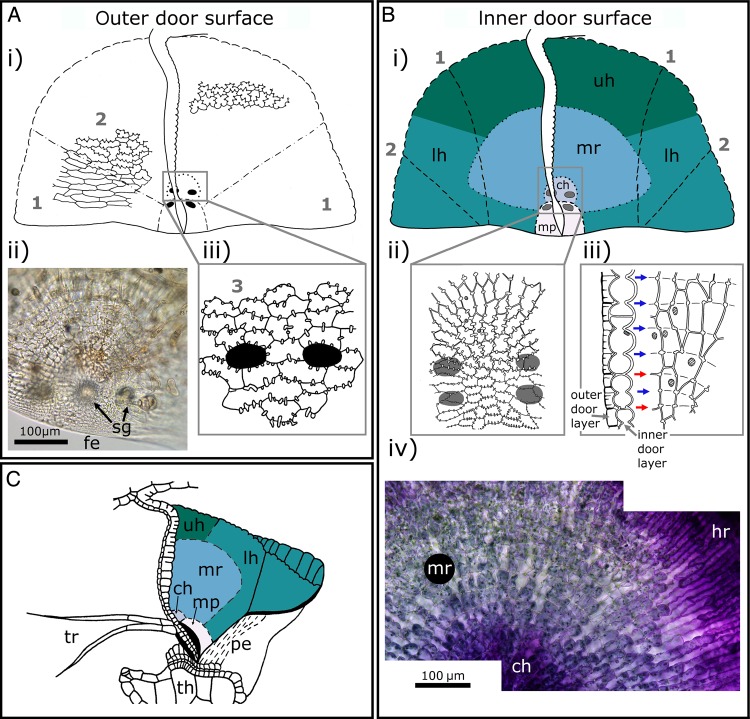
Cell types on and compartmentalization of the trapdoor. (Ai) Schematic drawing of the outer door surface of a trapdoor. The course of the anticlinal cell borders is indicated. For better orientation, a schematic longitudinal section of a trapdoor is also depicted in the middle. The dashed lines highlight the transition from areas of elongated cells with anticlinal borders that run in a wavy pattern without (or with only few) reinforcing ridges (indicated by (1)) to areas of cells with zigzag anticlinal borders and pronounced reinforcing ridges (2). (Aii) LM image of the central hinge on the outer surface of a trapdoor; note the free edge (fe) and spherically headed glands (sg). The trigger hairs are out of focus. (Aiii) Schematic drawing of the central hinge (3). The insertion points of trigger hairs are highlighted by black areas. The more or less isodiametric cells of the central hinge are much smaller (compared with (1) and (2)) and possess corrugated anticlinal borders with numerous reinforcing ridges. (Bi) Schematic drawing of the inner surface of a trapdoor, and various regions (upper hinge (uh), lateral hinge (lh), middle region (mr), central hinge (ch) and middle piece (mp)) highlighted with different colours. For better orientation, a schematic longitudinal section of a trapdoor is also depicted in the middle. The insertion points of trigger hairs on the opposite outer surface of the trapdoor are marked by grey areas (surrounded by a black line in Bi)). The dashed lines (1) separate the lateral areas of the trapdoor which curve outwards and (2) depict the areas that rest on the threshold when the trapdoor is closed. (Bii and Biii) Schematic drawings of the inner cell layer. (Bii) The patterns of anticlinal borders of cells in the central hinge and in the middle piece. The cells here are small, nearly isodiametric and possess numerous pronounced reinforced ridges. Several nuclei are indicated by shaded areas. The insertion points of trigger hairs on the opposite outer surface of the trapdoor are characterized by black areas (upper image) or by grey ellipses (lower image, for better visibility). (Biii) Courses of the concentric constrictions and of the anticlinal borders of cells of the inner trapdoor layer. The left sub-image depicts a longitudinal section of the trapdoor at its middle region. The right sub-image depicts the course of the anticlinal borders. The cut cell layer on the left side visible on the right sub-image corresponds to the cells of the longitudinal section of the left sub-image. Several nuclei are indicated by shaded areas. The cells of the inner layer of the trapdoor are regularly constricted in anticlinal direction, and the constrictions correlate mostly not (blue arrows) to the transversal cellular borders (red arrows). At the areas of constrictions (indicated by short lines), cell wall reinforcements can be found. (Biv) LM image of the inner layer of a trapdoor of *U. reflexa*, stained with toluidine blue. The hinge region (hr), middle region and central hinge are visible. (C) Schematic drawing of the positions of the door regions (highlighted by different colours) in a ready-to-catch trap of *U. gibba*. The trapdoor with trigger hairs (tr) rests on the pavement epithelium (pe) of the threshold (th). Note that the lateral hinge rests on the threshold with its outer surface. (Ai, Aiii, Bi–Biii and C) Modified from Lloyd (1932). (© Canadian Science Publishing or its licensors).

**Figure 9.**
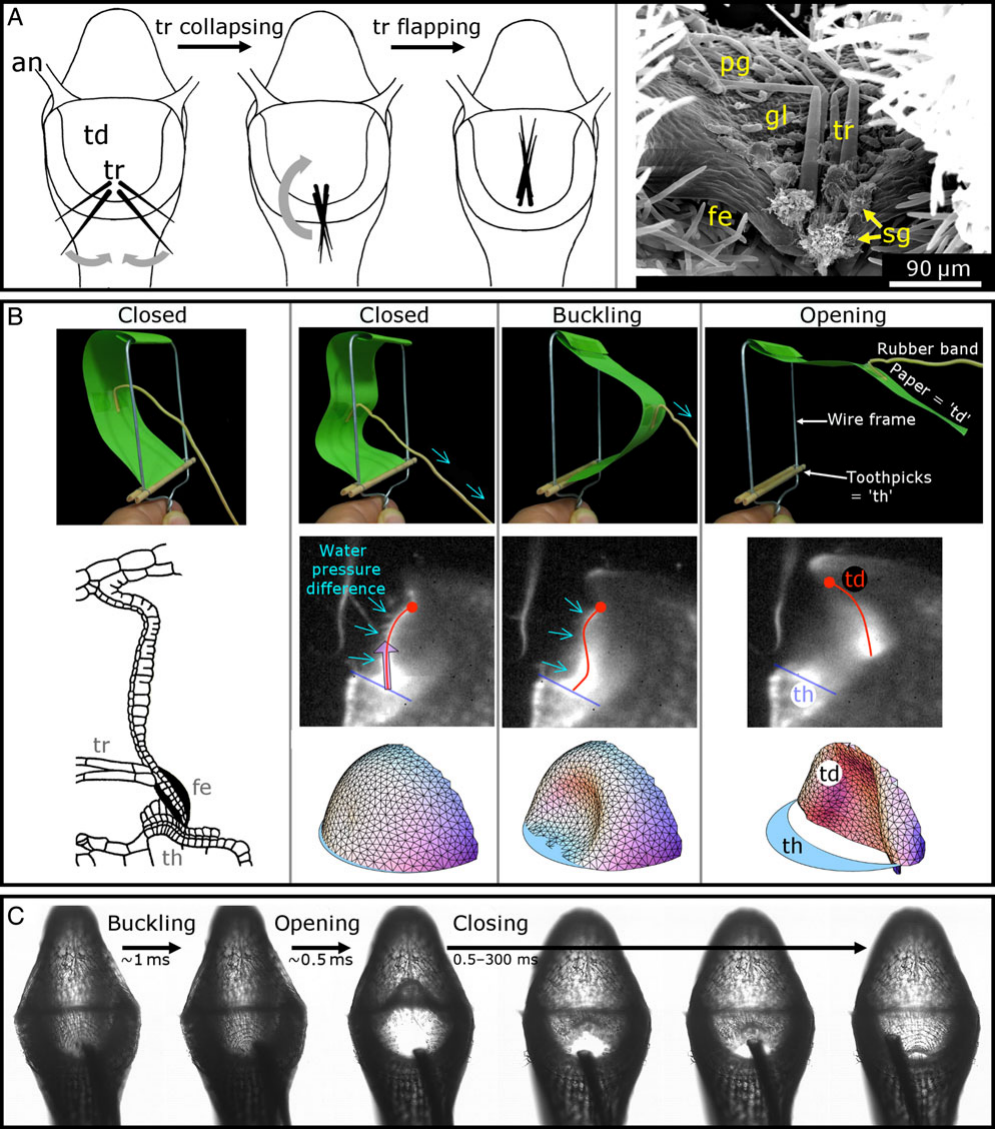
The trapdoor movement. (A) Frontal view of the trap entrance with ‘antennae’ (an). Schematic drawing of the collapsing and flapping motions of trigger hairs (tr) that are caused by the trapdoor (td) deformation (not shown). (B) Scanning electron microscope image of the outer trapdoor surface of *U. vulgaris*. The trapdoor has been manually pressed open at its lateral areas with a fine glass capillary. It can be seen that the trigger hairs have flapped onto the trapdoor surface similar as it is schematically depicted in the rightmost image in (A). Note also the free door edge (fe), spherically headed (sg) and pyriform glands (pg) as well as other glands (gl). (B) The different trapdoor movement steps are depicted. The upper images are photographs of the physical hand model proposed by Poppinga *et al*. (2013*c*). In this model, the ‘trapdoor’ does not possess a double curvature (as does the natural trapdoor), but nonetheless it can be regarded as a functional model. The schematic drawing on the left depicts the closed trapdoor in the ‘locked’ convex position and is modified from Lloyd (1932). (© Canadian Science Publishing or its licensors). The middle images are frames from high-speed recordings of a lateral view of a *U. inflata* trapdoor in ﬂuorescence laser sheet microscopy. The lower images are a dynamic simulation of the trapdoor. Both series (middle and lower images) are modified from Vincent *et al*. (2011*b*). Images situated on top of each other correspond to the same movement step (indicated as closed, buckling and opening). First, on the very left, the situation shortly after firing is shown. Only little force acts against the trapdoor because the pressure difference between the inside and outside of the trap is low. As the pressure difference rises (‘simulated’ by pulling a rubber band in the hand model), so does the force that acts on the trapdoor which becomes more and more mechanically sensitive. After triggering or spontaneously (critical pressure ‘simulated’ by further pulling the rubber band in the hand model), the trapdoor buckles inside (curvature inversion from convex to concave). In this position, the friction force on the threshold (th) (‘simulated’ by gaps between the toothpicks in the hand model) is low and the trapdoor cannot resist the force exerted by the water pressure difference any longer and swings open. (C) High-speed video frames (recording speed: 10 000 fps) of a frontal view of the trap entrance of *U. vulgaris* during suction. The door first buckles, then swings open and then recloses much slower.

A trapdoor closes the trap watertight (Figs 1D and E, 3A, and 4–8). Prey animals can trigger the trapdoor by touching trigger hairs on the outer door surface, which entails the second phase, comprising door opening in less than half a millisecond, trap wall relaxation and water (and thereby prey) influx due to the sudden increase of the trap volume (Merl 1922; Lloyd 1942; Sydenham and Findlay 1973) (Figs 4 and 9). In respect to trap movement duration, *Utricularia* is by far the fastest carnivorous plant (snapping in the Droseraceae *D. muscipula* (Venus flytrap) and *Aldrovanda vesiculosa* (Waterwheel plant) takes ∼100 ms (Forterre *et al*. 2005; Poppinga and Joyeux 2011; Poppinga *et al*. 2013*a*), and snap tentacle catapult movement in *Drosera glanduligera* takes 75 ms (Poppinga *et al*. 2012)). Vincent *et al*. (2011*b*) measured in *U. inflata* that the fluid inside the aspiration zone, which extends to a distance of up to ∼500 µm from the trapdoor, accelerates with up to 600 g and reaches a top speed of ∼1.5 m s^−1^, leaving prey animals in the vicinity of the entrance no chance to escape. The Reynolds number of the fluid reaches 900, indicating a laminar flow. Furthermore, prey often rotates and loops inside the trap in a motion away from the entrance (Fig. 4C), which is hypothesized to be crucial for retention of already caught prey, which otherwise might become flushed out of the trap. The suction-induced water swirl moves in opposite direction to the trap entrance and thus is unlikely to help in the door reclosing motion (see also the section about the trapdoor movement). Probably, the more or less triangular-shaped threshold and the overall lenticular appearance of the trap (see also the section about the functional morphology of traps of the *U. vulgaris* type) dictate the swirl direction, whereas rotation of the prey is likely induced by the shape of the animal.

The suction process can also be triggered manually by tools such as human hair, fine wire or needles (Czaja 1922; Merl 1922). After triggering, the elastic energy that is stored in the concave trap walls is converted into kinetic energy with the trap walls becoming convex (as seen from the outside) during the second phase (Fig. 4A). Due to the fact that piercing the trap, e.g. with a fine needle, leads to a stronger outward (convex) trap wall curvature than observed in an intact trap after firing (Fig. 4D–F), it can be speculated that the pressure difference does not become completely levelled by suction (Lloyd 1935). The kinetics of this plasticity effect of trap walls in opened traps has been described by Adamec (2011*d*). This is in concordance with our own investigations of high-speed videos of suction events which indicate that the trapdoor is already reclosed before the trap is fully inflated (i.e. before the trap walls are convex) (Fig. 4G). According to this, the reset force of the door that leads to closure (see also the section about the trapdoor movement) exceeds the force of the water inflow at a certain point, which effectively helps in preventing the escape of prey.

Caught prey suffocates due to anoxia inside the bladder (Adamec 2007*b*, 2010*b*). After being dissolved by digestive enzymes secreted by glands on the inner trap surface (see also the section about the functional morphology of traps of the *U. vulgaris* type), the nutrients can be absorbed by the plant. Both phases together form the repeatable ‘active slow deflation/passive fast suction’ sequence (Fig. 4A) found in traps of aquatic *Utricularia* species (Czaja 1922, 1924; Merl 1922; Withycombe 1924; Hegner 1926; Gibbs 1929; Lloyd 1929, 1932, 1935, 1936*a*, 1942; Kruck 1931; Sydenham and Findlay 1973, 1975; Meyers and Stricklert 1979; Sasago and Sibaoka 1985*a*, *b*; Friday 1989, 1991; Adamec 2011*c*, *d*, 2012; Singh *et al*. 2011; Vincent *et al*. 2011*a*, *b*).

The water is pumped out of the trap continuously and probably recirculates as soon as a given pressure difference is reached (Adamec 2011*d*). The outward flow is hypothesized to be compensated by an inward flow caused by trap wall permeability and/or trapdoor leakage (Joyeux *et al*. 2011; Vincent *et al*. 2011*a*). The time for resetting depends on the species studied (Meyers 1982) and varies with temperature (Withycombe 1924), trap age and, as for cut-off traps, the duration of storage (Sydenham and Findlay 1973). The trap of *U. vulgaris* is reset after ∼15–30 min. Traps are able to capture multiple prey animals one after another: Merl (1922) observed 13 prey capture events within 3 days in a trap of *U. australis*. Also, multiple prey animals can become captured with one suction swirl. In contrast to the snap-trap of the Venus flytrap (*D. muscipula*), no morphological change (i.e. growth) is required in the *Utricularia* trap for resetting and repeated trap firing (Vincent and Marmottant 2011).

### Spontaneous firings

Recent analyses (Adamec 2011*c*, *d*, 2012; Vincent *et al*. 2011*a*, *b*) showed that aquatic *Utricularia* traps also fire spontaneously without prey irritation after 5–20 h and up to 60 times in a 20-day period. It is hypothesized that, owing to the continuous process of water pumping, traps can generate a sufficient pressure difference for already (very) small mechanical perturbations (mechanical/thermal noise) causing trap firing (see also the section about the trapdoor movement). These spontaneous firings occur trap-individually in different, species-independent patterns: ‘metronomic traps’ fire regularly after more or less fixed time intervals, ‘random traps’ show temporally scattered suction events and ‘bursting traps’ display several rapidly succeeding firing events separated by variable time intervals. The ecological consequence of spontaneous firings for the plant is not yet clearly solved. Probably, they prevent material fatigue in the trap walls and, hence, traps to collapse. Moreover, a multitude of microorganisms (‘algae’, bacteria, protozoa, rotifers, etc.) can be found inside the traps that alone are not capable of triggering suction (Gordon and Pacheco 2007; Peroutka *et al*. 2008; Alkhalaf *et al*. 2009; Vincent *et al*. 2011*a*, *b*) but that could very well become accumulated by spontaneous firings. These organisms are in complex and not yet fully understood relationships with the plants. Recently, Koller-Peroutka *et al*. (2015) confirmed a prey biomass input by spontaneous firings, which adds to *Utricularia* nourishment.

## Functional Morphology of Traps of the *U. vulgaris* Type

**Figure 10.**
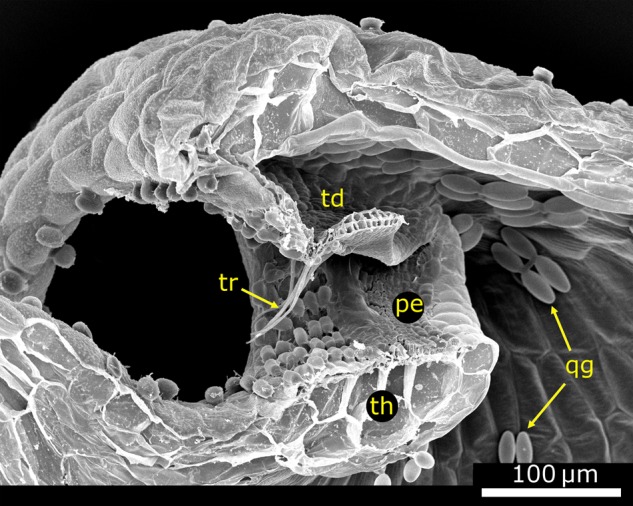
Scanning electron microscope image of a trap entrance with a small angle of ∼30° between trapdoor (td) and threshold (th). A longitudinal section of the trap entrance of terrestrial *U. longifolia* is shown; note the pavement epithelium (pe) and quadrifid glands (qg). For a comparison with aquatic *Utricularia* species, see Figs 6C and 7I.

In aquatic bladderworts, the traps typically constitute 10–50 % of the total plant biomass (Adamec 2007*a*, 2011*b*). In the following, the functional morphology of traps of the *U. vulgaris* trap type is described in detail. This trap type is characterized by an angle of ∼90° between the trapdoor and the threshold, as viewed in a longitudinal section (Fig. 5). This is in contrast to other, much less investigated trap types of non-aquatic species that are concisely described in a separate section at the end of this review (Fig. 10) (Lloyd 1935, 1936*a*, 1942).

### The trap body

The lenticular traps of species of *Utricularia* sect. *Utricularia* are typically between 0.5 and 6 mm in diameter (Taylor 1989; Adamec 2011*d*) (Figs 1–5). *Utricularia reflexa* may produce ‘giant’ traps that can reach up to 8 mm in size in culture (L. Adamec, pers. comm.).

#### The trap wall

The flexible trap wall mainly consists of two cell layers (Fig. 5B and D). Both the inner and outer walls are covered by a cuticle. The chlorophyll-rich cells are more or less quadrangular as seen in longitudinal section and elongated in transversal direction (Lloyd 1942). During the trap resetting phase, the lateral walls bend inside owing to the underpressure inside the bladder with the cells of the outer layer being compressed. The outer cell layer is thicker, which can be interpreted as a means for avoiding the trap to collapse. Vincent *et al*. (2011*b*) estimated the stiffness of the trap body to be in the range of 5–20 MPa, which is concordant with values measured for fully turgescent parenchymatous tissue (Niklas 1988; Speck and Vogellehner 1992; Speck 1994).

A single vascular strand (mostly phloem, sometimes also xylem) runs through the stalk into the trap body, here splitting up into two ‘branches’ that are arranged along the trap profile (Lloyd 1935, 1942). One branch runs along the dorsal line of the trap until it ends at the upper part of the trap entrance. The lower branch extends from the stalk to the threshold, there splitting up once more. From here on, both small vascular branches run laterally, then upwards and parallel to the trap opening and terminate in the ‘antennae’.

#### Glands on the outer trap surface

The spherically headed glands covering the outside of the traps and the outer surface of the threshold (Figs 5B and D, and 6) are comprised of a basal, middle and terminal cell (Thurston and Seabury 1975). They are of uncertain function. According to different authors, they might play a crucial role in pumping water out of the trap (Kruck 1931; Nold 1934; Sydenham and Findlay 1973) or in mucilage secretion (Barthlott *et al*. 2007). Fineran and Lee (1980) and Fineran (1980, 1985) state that these external glands, when being in the process of development, absorb solutes from the surrounding water, which later (after morphological changes of the glands) help in the water-pumping mechanism during the trap resetting phase. Owing to a negative periodic acid-Schiff stain, Thurston and Seabury (1975) conclude that these glands do not produce mucilage. These glands as well as those situated on the trapdoor (see Figs 7–9) or the substances released from them can both be stained by Toluidine blue (Fig. 6A and B).

#### Glands on the inner trap surface

Two types of glands can be found on the inner trap surface: two-armed glands that possess two terminal cells (bifids) and four-armed glands with four terminal cells (quadrifids) (Figs 5, 6C and D, and 7D–F, I and J) (Darwin 1875; Prowazek 1901). In some *Utricularia* species, these types of glands are replaced by other gland types (Taylor 1989). The epidermal basal cell is similar to the cells of the outer trap surface but slightly smaller. The compact middle cell typically resembles a disc or a dome. The intensely cutinized lateral walls with attached cell membrane suggest a barrier function for apoplastic transport into the terminal cell (Fineran 1985). Furthermore, the middle cell features noticeable cell wall inversions at the area of the outer transversal walls and upper half of the lateral walls (Fineran and Lee 1974; Fineran 1985; Płachno and Jankun 2004). The glandular secretory terminal cells of the bifids and quadrifids are regarded as the most modified cells found among plant trichomes (Fineran 1985), and the proximal regions of the ‘arms’ form a collective stalk with mechanically stabilizing, markedly thickened outer walls (Fineran and Lee 1975; Fineran and Gilbertson 1980). The two terminal cells of the bifids diverge at the distal part of the stalk, with each of the cells forming one arm of the gland. The four terminal cells of the quadrifids first form two opposite pairs that subsequently diverge. The angle between the arms and their general arrangement can be used for systematic purposes (Thor 1988; Cleal 1998; Doyle and Parnell 2003; Yang *et al*. 2009). The cuticle of the terminal cells either shows a discontinuous organization or ruptures during gland ontogeny (Fineran 1985).

Bifids are densely packed near the trap entrance on the inner surface of the trap body (Figs 5C and 6C). Every epidermal cell in this region develops such a gland. The two elongated terminal cells protrude into the trap interior being perpendicular to the inner surface of the trap. Quadrifids cover the rest of the inner trap surface (Fig. 5C). Each gland is surrounded by epidermal cells and hence separated from neighbouring glands. In contrast to bifids, the terminal cells of the quadrifid glands are arranged parallel to the inner trap surface.

The glands are responsible for pumping water out of the trap body, for secretion of digestive enzymes and for absorption of nutrients. Until now, it is not yet entirely understood what gland type fulfils which function. Czaja (1922), Merl (1922) and Withycombe (1924) suppose that quadrifid glands pump water out of the trap, a hypothesis that has not been rebutted until today (Skutch 1928; Kruck 1931; Nold 1934; Fineran and Lee 1975; Fineran and Gilbertson 1980). Kruck (1931) and Fineran and Lee (1975) postulate that the quadrifids are responsible for water transport together with the spherical glands on the outer trap surface. Fineran and Lee (1975) assume that the bifid glands help in the water-pumping process. Sydenham and Findlay (1975) and Sasago and Sibaoka (1985*a*) on the other hand postulate that the quadrifids serve the purpose of prey digestion and nutrient absorption. According to them, the pumping of water is performed by the bifids in cooperation with the glands of the pavement epithelium in the outer and middle zones that act as the outlets for the water outflow. This conclusion is due to the fact that water emerges exclusively next to the trap entrance (this observation was made with a trap resetting in paraffin oil) and as the bifid glands with their not cuticle-covered terminal cells are situated near this region. This separation of function between the two types of glands has not yet been verified but is considered as the current state of research in literature (cf. Juniper *et al*. 1989; Barthlott *et al*. 2007). Lloyd (1942) furthermore hypothesized that bifids also hinder caught prey animals to pass the threshold and to escape.

Although the hypothesis that the quadrifids are fully or partly responsible for prey digestion and nutrient uptake dates back to Darwin (1875) and von Goebel (1891), little is known about the process of digestion in *Utricularia*. Since the 1970s, cytochemical investigations of quadrifids revealed that protease (Vintéjoux 1974; Vintéjoux and Shoar-Ghafari 2005), acid phosphatase and esterase (Heslop-Harrison 1975) are secreted. Recent studies by Sirová *et al*. (2003) and Adamec (2010*a*) focussed on activities of extracellular enzymes (e.g. phosphatase).

### The trap entrance

#### Appendices at the trap entrance

At the trap entrance, several types of appendices occur which differ in number and structure and are of taxonomic importance (Taylor 1989; Reifenrath *et al*. 2006) (Figs 1D and E, 3A and D, 4D–G, 5, and 6A and C). Darwin (1875) termed the two multicellular and branched structures emerging from the upper edges of the trap entrance as ‘antennae’ and the filamentous, non-branched structures situated laterally on the trap entrance as ‘bristles’. Darwin used these terms because the overall shape of a trap reminded him of small aquatic crustaceans like *Daphnia*. With investigations on aquatic *U. vulgaris*, Meyers and Stricklert (1979) could confirm Darwin's hypothesis that ‘antennae’ covered with epiphytic algae (Prowse 1959; Guiral and Rougier 2007) enhance *Utricularia*'s capture success by guiding substrate-dwelling prey animals towards the trap entrance owing to their funnel-like arrangement (Figs 1E, 3A and 4G).

#### The threshold

The lower part of the trap entrance is constituted of a massive, collar-like tissue, which is bent upwards and termed threshold (von Goebel 1891) (Figs 1D, 3A, 4D–G, 5, 6, 7I, 8C and 9B). In longitudinal section, it resembles an upside-down triangle that merges into the trap wall (Figs 5 and 6). In its spatial dimension, it comprises four to five rows of parenchymatous cells and is surrounded by a layer of approximately isodiametric epidermal cells on each side, i.e. towards the interior and exterior of the trap (Lloyd 1942). The threshold surface is undulated owing to varying sizes of the epidermal cells and of the parenchymatous cells underneath (Fig. 6B and D–F). At the lateral transition area connecting the threshold and the trap wall, the epidermal cells increase in size and merge into the inner and outer cell layers of the trap wall (Fig. 5B). At this transition zone, the trap wall is relatively thin by which deformation of the threshold owing to the underpressure-induced deformation of the trap walls is avoided. The threshold mechanically stiffens the entrance and reduces deformation during the resetting phase and during suction. Independent of the physiological trap condition, it maintains its geometry and helps ‘framing’ the door, which is sensitive to mechanical perturbations (see also the section about the trapdoor movement).

The threshold surface is slightly convex and can be divided into an outer, middle and inner region (Lloyd 1942; Juniper *et al*. 1989) (Fig. 6D–F). The outer region represents part of the entrance corridor and comprises epidermal cells, which sometimes carry stalked glands (Figs 5B, and 6D and F). The inner region of the threshold also consists of epidermal cells and forms a nose-like structure that extends into the trap (Figs 6B, D and F, and 7J). It is considered as being part of the inner trap surface. The middle region (Fig. 6D–G) is constituted of tightly packed, short glands forming a specialized glandular tissue on which the door rests. According to its visual appearance—the glandular surface resembles a cobble stone pavement—von Goebel (1891) termed it the ‘pavement epithelium’. Alike the stalked glands that can be found on the trapdoor and in the entrance zone, those of the pavement epithelium consist of three cells (epidermal basal cell, endodermal middle cell and glandular terminal cell) (von Goebel 1891; Lloyd 1942; Thurston and Seabury 1975; Fineran 1985). During ontogeny, the cuticles separate from the terminal cells and become shed.

According to Lloyd (1932, 1936*a*, 1942), three pavement epithelium zones (outer, middle and inner zone) can be distinguished by gland morphology (Figs 6E–G and 7J). These three zones are all parts of the middle region of the threshold surface. The outer zone is characterized by glands with cuticles, which are bloated like balloons. Cuticles from glands of the middle zone become shed but stick together and with the cuticles of glands of the outer zone. The resulting filigree, transparent, membranous structure is termed velum (Lloyd 1929) (Figs 5B, 6C–F, and 7I and J). It consists of two parts: the cushion-like structure of connected balloon-like cuticles that run along the whole outer region and, connected to this cushion, the membrane emerging from cells of the middle region. The putatively ‘sticky’ (see below) velum is hypothesized to cling to the free edge of the closed trapdoor and hence to play a mechanical role in trapdoor movement and to help for maintaining the trap sealed watertight (Lloyd 1935, 1942; Broussaud and Vintéjoux 1982; Fineran 1985). Kurz (1960) argues that the velum consists of swollen cell membrane without cytoplasm, whereas Lloyd (1942) and Heide-Jørgensen (1991) suppose the velum to consist of a stretched protocuticle. The gland heads in the middle zone possess a soft surface (owing to the shed cuticle) in which the lower free edge of the trapdoor can subside. The inner zone of the pavement epithelium is broadest in the middle of the threshold and becomes thinner more laterally, as viewed from above (Fig. 6G). Here, the glands are larger and less densely packed as in the two other zones. Their cuticles also detach from the cells but mostly rupture. It is still not solved which mechanism leads to cuticle detachment, but probably it may be due to cell exudation. Glands on the pavement epithelium are supposed to secrete a polysaccharide mucilage (Cheema *et al*. 1992), which helps for sealing the trap entrance watertight (Withycombe 1924; Fineran 1985) and/or for lubricating the entrance to ensure a smooth door movement (Lloyd 1942).

The threshold exhibits two bumps on its surface: a larger elevation comprising the outer and middle zone of the pavement epithelium and a more shallow elevation at the inner zone (Fig. 6E and F). The cavity between these two bumps acts as a furrow for the free door edge in its closed state (Figs 6F and 8C). At the bottom of this cavity, there is the transition between the middle zone to the inner zone of the pavement epithelium, and the door edge rests on the flexible gland heads of the middle zone. In a ready-to-catch trap, the outer door edge surface presses against the slopy edge of the anterior bump. An inward swinging (opening) of the trapdoor is prevented by a second, more shallow bump. Despite the water pressure acting on the door, the door edge cannot pass this bump without previous trigger-induced deformation and slight displacement from the surface (see also the section about the trapdoor movement).

#### Glands at the trap entrance

The lateral and ventral inner surface of the trap entrance, near to the pavement epithelium, is covered with long-stalked pyriform glands resembling those on the upper part of the middle region on the door (Figs 5B and D, 6B–D and F, and 7C, E and I). The lengths of their basal cells decrease in direction to the trapdoor. Darwin (1875) postulated that these glands absorb substances, alike the quadrifid glands covering the inner trap body surface, which are released during the process of digestion. In addition, they were suspected to attract prey animals (Cohn 1875; Büsgen 1888; von Goebel 1891; Kurz 1960). Thurston and Seabury (1975), by investigating *U. biflora*, attributed mucilage production to these glands as well as to the stalked glands on the trapdoor and to the bifid and quadrifid glands, owing to positive periodic acid-Schiff staining and due to the gelatinous and filamentous substances sticking on the terminal gland cells. In contrast, Fineran and Lee (1980) could not detect mucilage secretion by the stalked glands in the entrance zone of terrestrial *U. dichotoma*. Sydenham and Findlay (1975) hypothesized that the water taken up by the bifid glands is released to the trap exterior by the stalked glands of the entrance zone or by glands of the pavement epithelium, but the fact that not all *Utricularia* species possess stalked glands in the entrance zone makes this assumption unlikely (Fineran 1985).

#### The trapdoor

The semi-circular trapdoor is a ∼20–40 µm thick flap-like structure constituted of two cell layers which closes the trap mouth watertight (Figs 5, 6B–D and F, 7, and 8C). The door is fixed laterally to the trap wall and to the upper part of the trap entrance along a curved arch, hence retaining a free lower edge (Fig. 7A and C–F). The free edge has a pointed tip which can be seen in longitudinal section (Figs 6C, D and F, and 7B and I) and is thicker in the middle part of the door than in its lateral parts. The door shows an outward curvature when it is closed and when the trap is ready to catch (Figs 8C and 9B). The outward curvature is *inter alia* due to the fact that the free edge of the trapdoor is longer than the contact area on the pavement epithelium (Fig. 6G) (see also the section about the trapdoor movement). Owing to the collar-like appearance of the threshold, the angle between the free trapdoor edge and the pavement epithelium changes along this contact area. In the middle area, the door rests in an approximate right angle on the pavement epithelium, which changes to increasingly acute angles in the more lateral regions. At the outermost parts, the surfaces of the trapdoor are in contact with the pavement epithelium (Lloyd 1942).

#### Regions on the trapdoor

According to Lloyd (1942), the trapdoor can be compartmentalized into four regions (Figs 7B, D, F and H, and 8B and C): the hinge region, the middle region, the central hinge and the middle piece. The hinge region comprises a broad zone along the connection between the door and the trap wall, thereby surrounding the middle region, the central hinge and the middle piece. It can be subdivided into two mechanically relevant parts: one upper hinge and two lateral hinges. When the trapdoor is closed, the upper hinge shows an inward curvature (Figs 7B and 8C), which is strongest in the middle of the trapdoor and is most pronounced in the set condition. The lateral hinges comprise the zones at which the door moves back and where the outer door surface partially rests on the pavement epithelium. The central part of the door (*inter alia* comprising the middle region) shows an outward curvature in contrast to the upper hinge, i.e. it is convex when seen from the outside (Figs 7B and 8C). On the middle of the trapdoor's lower edge, there is a circular area, the central hinge, where two of the four trigger hairs are located. Here, the trapdoor is comparably thin. Below the central hinge, the middle piece is located (Fig. 7H). Here, the lower trapdoor edge is thick and stiff, and on its outer surface, the other two trigger hairs are located. The central hinge, the middle piece and the areas on the lateral hinges that are situated below the middle region show a convex curvature, when seen from the outside in the set posture, and have the approximate shape of the quarter of an ellipsoid surface (Fig. 8C).

The inner layer of the trapdoor consists of elongated cells that have been described to function as compressive bellows (Lloyd 1942; Juniper *et al*. 1989) and are radially arranged around the central hinge (Figs 7D, F and H, and 8B). Constrictions of these cells appear as patterns of circular lines in the middle region of the trapdoor and presumably increase its flexibility in radial direction, hereby acting as prefolds for channelling the reproducible door opening and closing (Lloyd 1942; Juniper *et al*. 1989; Vincent *et al*. 2011*b*) (see also the section about the trapdoor movement). The cells of the hinge region along the trap wall are not constricted and might act as a spring structure for door closure. The smaller cells of the outer door layer are not distinctly compartmentalized (Figs 7C and E, and 8A). Vincent *et al*. (2011*b*) also noticed lateral trapdoor folds (Fig. 7C–G) that are hypothesized to add displacement space during suction of prey by unfolding, to ease and channel the in- and outwards bending of the trapdoor and to allow for positioning the relatively long free trapdoor edge on the pavement epithelium permitting a watertight closure.

#### Histology of the trapdoor

From an anatomical point of view, the trapdoor is a continuation of the trap wall and hence analogically constituted of two cell layers (Fig. 7B). The relative thickness of the layers varies with the function of the respective door region. In many parts (hinge region and middle piece), the outer cell layer is up to three times thinner than the inner layer. Exceptions are the central hinge and the middle piece where both layers are approximately of equal thickness. Large differences in thickness of the two cell layers mainly occur in door regions that take mostly part in the door movement and hence must be flexible. At the central hinge, the trapdoor is thinnest (∼13–16 µm in *U. vulgaris*).

The inner and outer cell layers of the trapdoor also differ according to their general structure. The outer cell layer consists of tabular cells with anticlinal borders running in a wavy or zigzag pattern (Büsgen 1888; Lloyd 1932, 1942) (Fig. 8A). The curves and corners of the borders possess, according to the respective region of the trapdoor where they are situated, more or less pronounced reinforcing ridges, which prevent collapsing of the respective cells during deformation. In the upper hinge, and partly also in the middle piece and in the lateral hinges, the cells are more or less isodiametric and the anticlinal borders also follow a zigzag pattern and possess reinforcing ridges (Cohn 1875; Lloyd 1932, 1942). In direction to the outer surface of the lateral hinges, the corners become more and more roundish and the anticlinal borders adapt to a wavy pattern. The cells here are transversally elongated, do not possess reinforcements and run parallel to the free door edge. The outer cells of the central hinge, however, are very small, nearly isodiametric and feature many reinforcing ridges that form a complex pattern. In the middle piece, the outer cell layer also features very small, isodiametric cells with reinforcing ridges forming a bilateral symmetric pattern (Lloyd 1932). Hence, the outer door regions, e.g. the hinge region, which become strongly deformed during the door movement, are characterized by cells with zigzag anticlinal borders and small reinforcing ridges, whereas the stiffest region (middle piece) exhibits the thickest cell walls and a multitude of noticeable reinforcing ridges.

The inner cell layer of the trapdoor (Fig. 8B) in the middle and hinge regions consists of elongated cells that run radially. Their size increases with the distance to the central hinge. The anticlinal borders are thin, run in a zigzag or wavy pattern and possess many reinforcing ridges. The periclinal walls facing the trap inside are, in contrast to the anticlinal borders, much thicker. Moreover, the inner cells are constricted in a constant fashion perpendicularly to the inner door surface. These constrictions run along several cells, seldom conforming to the transversal cell borders. Ekambaram (1916) and Czaja (1922) hypothesized that the constrictions follow the transversal walls and described the cells as isodiametric (Fig. 8B). Hence, the periclinal cell walls are characterized by several convex curvatures with cell borders that may run across the apex of these bulges. At the zones where the constrictions meet the anticlinal borders, reinforcing ridges or plates are developed. In the inner part of the middle piece, the constrictions run concentrically around the central hinge. Irregularities only exist near the longitudinal axis and near the free edge of the trapdoor where the constrictions furcate. In the upper and lateral hinges, the constrictions are less noticeable than in the middle region. The cells of the central hinge and of the middle piece are very small and isodiametric, and the inner cell walls are characterized by a multitude of reinforcements. The periclinal cell walls of the central hinge also feature constrictions, which in contrast do not run only concentrically but also perpendicularly in radial direction. In the middle piece, there are no constrictions, but the periclinal borders are much thicker than in the other door regions (Lloyd 1932, 1942).

Due to the above described structuring, the two-cell-layer-thick trapdoor regions possess different mechanical bending and stretching properties. Despite both being turgescent, only the cells of the inner layer deform noticeably in the middle and hinge regions (Lloyd 1942; Juniper *et al*. 1989). Such a bilayer structure further entails that the door can only move towards the trap inside. A manual outward pressing leads to a rupture of the trapdoor because it is tightly framed in the trap entrance. When the trapdoor is cut-off from the entrance, it will instantly flip outwards and will only reset by plasmolysis. According to this scenario, the door most likely is under tension by turgor in its resting position. Presumably, the structural differences in the various trapdoor regions contribute to the tension that helps keeping the door in its resting position on the threshold and to enforce the door closing after prey capture (see also the section about the trapdoor movement).

#### Glands on the trapdoor

The outer door surface is covered with a multitude of glands of different types. In the upper part of the middle region, there are situated several long-stalked, three-cellular glands with pyriform terminal cells (Figs 5, 6A–C, and 7B, C, E and I). They form a broad, band-like seam along the transition zone between the upper hinge and the middle region. Near the free trapdoor edge three-cellular stalked glands with spherical terminal cells are found (Figs 7C, E, H and K, and 8A). The stalks of these glands are much shorter than those found in the pyriform glands and their terminal cell is much larger in relation to the stalk diameter. These short-stalked glands form a row that runs parallel to the free trapdoor edge underneath the trigger hairs and reaches up to the lateral hinges. They are arranged in a manner alternating to the trigger hair bases, and the central gland is larger than the rest. Between the trigger hairs and the long-stalked glands, the middle region of the trapdoor often is covered by two-armed, sessile glands (Fig. 7B, C and E; Darwin 1875). The function of these glands is not yet known, and it is speculated that they may play an important role in either absorption or prey attraction (Darwin 1875; Büsgen 1888; Meierhofer 1902; von Luetzelburg 1910).

#### Trigger hairs

Mostly four stiff, pointed and long trichomes protrude from the central lower part of the trapdoor (central hinge and middle piece) (Figs 1E, 6C and D, and 7A–C, E, I, K and L). These trigger hairs are arranged as stacked pairs in the form of a rectangle or a slightly shifted trapezoid. They are constituted of three to up to five elongated cells with an increasing cell length from the basal cell to the top (Lloyd 1942). They are inserted obliquely to the door and anchored in the outer trapdoor cell layer with a bulged basal cell (Meierhofer 1902; Merl 1922; Lloyd 1942) (Fig. 7L). Some *Utricularia* species (not of *U.* sect. *Utricularia*) are lacking trigger hairs but feature stalked glands (e.g. *U. cornuta* and *U. purpurea*) or other types of trichomes (Taylor 1989).

Mechanical stimulation of one of the trigger hairs leads to trapdoor opening and suction of water, independent of the direction of the stimulus. According to Lloyd (1932), manual triggering is ‘easier’ when performed laterally or from above. Two hypotheses exist on how the trigger hairs work. The mechanical hypothesis postulates that the hairs act as levers (Czaja 1922; Merl 1922; Lloyd 1932, 1935, 1942). A trigger hair bending deformation owing to contact by prey is transduced to the middle piece of the trapdoor which also deforms, resulting in a slight displacement of the free trapdoor edge on the pavement epithelium and causing it to pass over the barrier bump (see also the section about the trapdoor movement) (Fig. 6E and F). The trapdoor can no longer resist the water pressure and opens. According to the physiological hypothesis, the trigger hairs are analogues to the sensory hairs of the Venus flytrap (*D. muscipula*) and waterwheel plant (*A. vesiculosa*) (both Droseraceae) (Brocher 1912; Ekambaram 1924; Withycombe 1924; Kruck 1931; Diannelidis and Umrath 1953; Sydenham and Findlay 1973; Broussaud and Vintéjoux 1982). In this scenario, a mechanical stimulus on the hairs results in the generation of an electrical signal that is transduced over the trapdoor and leads to cell contraction (Kruck 1931), respectively turgor changes (Ekambaram 1924), followed by trapdoor deformation and finally trapdoor opening. None of these hypotheses could be verified until now, and Juniper *et al*. (1989) suppose that both might hold true for at least some *Utricularia* species. Adamec (2012) showed that diethylether, a membrane ion channel inhibitor, and sodium azide, a cytochrome oxidase inhibitor, as well as cold temperature (2 °C) negatively influence the physiological processes involved in trap resetting but not triggering, which gives support to the mechanical hypothesis.

## The Trapdoor Movement

The suction of water and prey relies on a trapdoor that on the one hand reliably seals the trap watertight (in the resting position) and on the other hand performs an ultra-fast and reversible opening and closing movement (during the suction process). The structural prerequisites described allow for the complex motion pattern of the trapdoor, which is described in the following.

### Trapdoor position before suction

In a ready-to-catch trap, the force exerted on the door due to water pressure difference is levelled by the friction force exerted by the pavement epithelium on the free trapdoor edge. The outwards curved (convex) door is in a metastable state (‘unstable equilibrium’, cf. Brocher 1912) (Fig. 8B). Its free edge becomes firmly pressed against the middle zone of the pavement epithelium (Fig. 6F and G). The free edge of the trapdoor rests with its outer surface on the bulged outer zone of the pavement epithelium and its lateral zone rests on the threshold (Fig. 8C). Beneath the five short-stalked glands on the trapdoor, the velum closes the gap between trapdoor and threshold along the connection area (Lloyd 1936*a*) (Figs 6F and 7I). It is still unclear how the lateral folds are positioned in the closed door in a ready-to-catch trap as well as how they unfold during the opening. Furthermore, the question of how notch stresses are (probably) structurally avoided in these folds remains to be answered.

### Trapdoor opening and closing movement

The tiny traps of bladderworts and the high speed of their trapdoor movement beyond human visual perception impeded detailed analyses of their movements for a long time. However, the early investigations by Ekambaram (1916, 1924), Sydenham and Findlay (1973) and especially Lloyd (1932) are quite noteworthy. Despite their mostly hypothetical character, the results presented are close to the much later described kinematics derived from high-speed cinematographic analyses. In detail, Lloyd (1932) states that touching the trigger hairs results in a slight deformation of the middle piece of the trapdoor and a concomitant displacement on the threshold. Based on the observation that the free door edge is longer than the threshold (Lloyd depicts a length ratio of 110 : 100), he concludes that it must deform in its middle zone (in the area of the middle piece) to be able to pass the inner bulging of the pavement epithelium. By using a bent wire, he physically simulated the deformation of the free trapdoor edge and showed that its curvature completely inverts during trapdoor opening. Moreover, he states that the trigger hairs collapse and flap against the outer trapdoor surface (Fig. 9A). The closing movement of the trapdoor after prey capture starts when the influx of water flow diminishes and is owing to the intrinsic mechanical properties of the trapdoor.

Quite recently, high-speed cinematography allowed for recording and analysing the trapdoor kinematics in full time resolution (Vincent *et al*. 2011*b*). The trapdoor first inverts its curvature after triggering (with the trap still being closed) (Fig. 9B). This quick deformation is a snap-buckling process (or snap-through transition) that starts in the thickened middle piece of the trapdoor below the central hinge and is concomitant with converging trigger hairs flapping against the trapdoor (Fig. 9A). The trigger hair movement is due to the morphology of the trapdoor in the areas of the trigger hairs insertion. Here, the free trapdoor edge is thickened, which is visible in form of bulges on the outer and inner trapdoor surface (Fig. 7H and K). These thickenings are interrupted in longitudinal direction by an area in which the trapdoor is very thin. At this special area, the initial buckling (after triggering) causing trigger hair movement takes place. The buckling proceeds all over the trapdoor until the door inverts its curvature completely. After this fast snap-buckling, the trapdoor swings open in ∼0.5 ms (as measured on average for *U. inflata* and *U. vulgaris*) and then recloses by regaining its original curvature (unbuckling and concomitant unfolding of the trigger hairs) within 0.5–300 ms (Fig. 9). In a figurative sense, the first step after triggering (trapdoor buckling) can be regarded as an ‘unlocking’ of the trapdoor because once in the buckled position (concave trapdoor curvature), it cannot resist the water pressure any longer and swings open. As long as the suction flow force is high enough (i.e. higher than the intrinsic reset force of the trapdoor), the trapdoor remains open. The closing of the trapdoor is often much slower than its opening as it is solely driven by the reset force of the trapdoor and is presumably not supported by the release of elastic energy stored in the trap body and the water swirl inside the trap (see also the section about the functional principle of the traps). The trapdoor, which does not move like an articulated flap but rather than an entirely deformable elastic thin shell, was computationally finite element modelled by Joyeux *et al*. (2011) (Fig. 9B). These simulations confirmed the experimentally observed complex kinematics, i.e. a rapid curvature inversion of the trapdoor before opening. Llorens *et al*. (2012) proposed a dynamical model incorporating the whole trap. A simplified physical door hand model for educational purposes was developed by Poppinga *et al*. (2013*c*) (Fig. 9B). Interestingly, snap-buckling as a movement principle has been initially described for the trapping motion in the Venus flytrap (*D. muscipula*) (Forterre *et al*. 2005), where a one-way buckling leads to the fast trap movement (whereas in *Utricularia* both buckling and unbuckling are essential for a trap to function).

### Trapdoor position after suction

During prey capture, a partial pressure equilibration takes place between the trap inside and outside (see also the section about the functional principle of the traps). Afterwards, the water pressure acting on the trapdoor is lower than in the ready-to-catch state. Hence, the trapdoor takes a position which is dictated more by its architecture than by water pressure, and therefore, its shape is more or less convex (as seen from outside). Its lower free edge also rests on the pavement epithelium, but closer to the outer border than in the ready-to-catch trap state. Despite the lower pressure difference between the trap inside and outside, the relaxed trapdoor and the velum both close the trap body watertight (Lloyd 1936*a*).

### Implications of the trapdoor buckling scenario

By the above-mentioned buckling scenario, the mechanical reasons underlying spontaneous firing as well as the triggering mechanism can be (partly) explained. With the traps continuously pumping water out, the internal/external trap pressure difference comes close to a critical pressure for spontaneous trapdoor buckling and suction, which may happen owing to thermal or mechanical noise. Hence, the mechanical sensitivity of the trap increases with the pressure difference. Shortly after firing, the difference is not high enough so that mechanical perturbations on the door do not lead to buckling and suction (cf. Adamec 2011*c*). After ∼15–30 min, the pressure difference is high enough again, so that mechanical perturbations (together with the water pressure acting on the door) entail trapdoor buckling and trap firing. This scenario supports the hypothesis of mechanical trapdoor triggering described in the chapter about the trigger hairs, where a minute trigger hair displacement (which acts as a lever) leads to a slight trapdoor deformation that entails buckling. The physiological hypothesis is herewith not yet ruled out, as the inactivity of traps shortly after firing could also represent a physiological refractory period. Finally, when the pressure difference reaches a critical value of ∼0.155 bar (as calculated by Vincent *et al*. 2011*a*), the trapdoor buckles spontaneously and the trap fires. However, by none of these hypotheses, the burst firing observed in some traps (see also the section about the functional principle of the traps; Vincent *et al*. 2011*a*) can be explained. Probably, this firing type is caused by a variable position of the trapdoor edge after each firing on the sealing pavement epithelium, which could explain why a different critical underpressure is needed for firing of the same trap in the course of time.

Vincent *et al*. (2011*b*) proposed a general architectonical ‘law’ for suction traps, taking the trapdoor buckling scenario into account. Too thick and stiff a trap body would not deform enough and would suck only a little amount of water, and too soft a trap body would be too slow during the passive fast suction phase. For an optimized trapdoor to open at a stage of maximum trap deflation (so that the trap can suck a maximum amount of water and prey), it must be considerably thinner than the respective trap body. The consideration of such a door-to-body-thickness ratio may be helpful to estimate trapping behaviour in *Utricularia* species where the suction mechanism is doubtful owing to morphological peculiarities, e.g. due to having exceptionally thick trapdoors (Reifenrath *et al*. 2006). However, thick and stiff traps have recently been found in some Australian *Utricularia* species from the *Pleiochasia* section (Płachno *et al*. 2015).

## A Comparison of the Different Trap Entrance Types in *Utricularia*

Knowledge on trap function and especially trapdoor movement of non-aquatic *Utricularia* species is still limited, because the respective traps are difficult to investigate due to minor size and a multitude of obstructing appendages around the trapdoor. The most detailed examinations on the entire genus *Utricularia* were conducted by Lloyd, who distinguished between short and long tubular trap entrance types (Lloyd 1936*a*, 1942) and defined in the latter two variants, in which the shape of the relaxed and curved trapdoor either can be circumscribed by one continuous bend or by two bends. In the short tubular trap entrance type, the angle between the trapdoor in its relaxed posture and the pavement epithelium measures ∼90°. This is referred to as the *U. vulgaris* trap type, which is described in detail in this review. In contrast, long tubular entrance traps are narrower and exhibit a narrow angle of ∼30° between the trapdoor and the threshold (Fig. 10). Conspicuously, species of the *U. vulgaris* trap type are aquatics, whereas many non-aquatic species belong to the narrow-angled type. However, Lloyd did not find a consistent correlation between entrance types and the sections or species, respectively. Since the morphological traits of the aquatic section *Utricularia* have been described in detail in this review, the focus in this section is on the long tubular entrance type, which is common in non-aquatic species, such as *U. dichotoma*, *U. caerulea* and *U. cornuta*. In this type, the trapdoor is longer (from the hinge region to the free edge) than broad (length of the free edge), thicker than in the *U. vulgaris* type and possesses a massive middle piece. In the set position, these trapdoors are concave, in contrast to the convex trapdoors found in *U. inflata* and *U. vulgaris*. Due to the underpressure inside the traps, the trapdoor (which is in an oblique angle towards the trap lumen) is pressed on the threshold, which is aided by the stiffness of the lateral hinges and by a concavity. The trap is additionally sealed by a velum (Lloyd 1936*a*). Upon his detailed anatomical examinations in *U. bisquamata*, Lloyd (1936*b*) concluded that along the axis of the trapdoor, there is a very narrow region of greater flexibility, so that during opening, a momentary sudden longitudinal bending occurs, leading to a reversal of the curvature. In the relaxed position, the trapdoor is convex, the angle between trapdoor and pavement epithelium is increased and the trapdoor becomes watertight again. Over the course of resetting, the trapdoor gradually returns to the concave set position. Many non-aquatic species, for example the entire section *Pleiochasia*, are devoid of trigger hairs (Lloyd 1942; Taylor 1989; Reifenrath *et al*. 2006), which leaves the question how they can be stimulated. The phylogenetically early-branching species *U. multifida* possesses a very thick trapdoor, so that Reifenrath *et al*. (2006) suspect that it cannot perform low-pressure suction movement altogether (see also the section about the trapdoor movement). Due to the extremely rapid trapdoor movement of bladderworts and the lack of modern equipment, Lloyd was not able to perform a detailed investigation on the exact opening procedure in the long tubular entrance type. Surprisingly, despite the high structural differences within the trap entrances, the functional principles of the trapdoor movement in non-aquatic *Utricularia* have not been subject to investigation since.

## Promising Aspects for Future Studies

Although we already have a detailed knowledge on the function of the bladderwort traps, there remain numerous intriguing questions of which some are decades old and still remain unanswered. First, since the comprehensive works by Lloyd in the 1920s–40s, there have not been conducted detailed analyses of the trap and trapdoor motions in *Utricularia* species outside of section *Utricularia* (see also the foregoing section). Such species possess different door-to-threshold angles, tubular entrances, different door-to-body-thickness ratios, other structures for prey attraction, trigger hairs and they probably have divergent prey spectra (cf. Reifenrath *et al*. 2006; Albert *et al*. 2010). We believe that experimentation in the laboratory, as well as in the field, with this highly diverse genus would lead to a multitude of new insights on trapping mechanisms, especially in regard to trap architecture and probably prey diversity.

One of the oldest questions is how does the triggering work? The trigger hair sensory cells in the Venus flytrap (*D. muscipula*) are very well investigated and are characterized by spirally running, concentric endoplasmatic reticulae (ER) at the respective poles, which surround large vacuoles filled with phenolic substances. Williams and Mozingo (1971) hypothesize that these ER complexes function as pressure transmitter by causing a release of the phenolic substances upon mechanical deformation. Probably, investigations on trigger hairs of *Utricularia* species using a transmission electron microscope (TEM) will shed light on similar or different cellular architecture enabling or excluding a physiological sensitivity. Furthermore, electrophysiological experiments (e.g. electrical irritation, measurements of the ion distribution with microelectrodes and of turgor pressure) could help elucidating the question if triggering of the trap is purely a mechanical process or not (cf. Adamec 2011*a*). Moreover, detailed microscopic studies of the middle piece and more generally on the lower free edge of the trapdoor with high-speed cameras capable of high physical (pixel) resolution combined with high temporal resolution (recording speed >10 000 fps) should allow for comparative analyses of the kinematics of the trapdoor motion during spontaneous (un-triggered) firings and in manually triggered traps. With such a detailed analysis, it might be possible to determine whether the displacement/initial movement of the trapdoor always follows the same pattern (which would speak for the involvement of an electrophysiological step) or whether small differences occur (which would speak for the mechanical lever theory).

Furthermore, direct investigations *in situ* on spontaneous firings, and how they might be ‘controlled’ by certain environmental factors, have not yet been conducted. With mobile and waterproof time lapse cameras, the temporal patterns of spontaneous firings under natural conditions can be monitored, and the intake of biomass per trap could be quantified.

Until now, the process of water pumping out of the trap is not yet fully understood. Tracer experiments with heavy water or low-molecular/fluorescent dye could help in elucidating the pathway of water out of the trap. Moreover, the function of the glands on the trap entrance, on the trapdoor and on the trap body is still unknown. Detailed anatomical analyses (e.g. with TEM) and selective staining of the substances secreted are necessary to gain further knowledge of these structures.

Underpressure measurements in a high temporal resolution are indispensable for determining the physical boundaries for deflation, door buckling, successful prey capture and spontaneous firings. Non-invasive measurements (optical quantification of changes of trap width, observation of air bubbles inside the traps) are still very difficult to perform, but are in principle possible.

A challenging task is also the elucidation of how the lateral flaps on the free trapdoor edge take part in the overall trapdoor movement, i.e. to answer the question whether they provide additional space by unfolding into the trap. For building up the underpressure inside the trap, which is necessary for trap firing, the traps must remain unharmed, so that it is not possible to record a manually evoked trapdoor motion in a sectioned trap. One would need fine borescopes or similar optical devices inserted into the traps combined with a very light-sensitive high-speed camera to record the trapdoor motion from inside the trap. Probably, the exact position and arrangement of the flaps could be analysed microscopically if the trap could be fixed in the ready-to-catch state by chemical or other means.

Last but not least, the suction trap of *Utricularia* could act as a role model for biomimetic suction devices capable of repeatable ultra-fast collection of small amounts of liquid.

## Sources of Funding

We thank the German Federal Ministry of Education and Research (BMBF) (funding directive BIONA, 01RB0806) for long-lasting financial support. The current work is funded by the Innovationsfonds Forschung of the University of Freiburg and by the German Research Foundation within the Cooperative Research Center CRC 141 ‘Biological Design and Integrative Structures’.

## Contributions by the Authors

All authors were involved in literature research and reviewing. S.P., C.W. and A.S.W. recorded scientific figures and wrote the first draft of the manuscript.

## Conflict of Interest Statement

None declared.
